# Wild Edible Plants Used in Dalmatian Zagora (Croatia)

**DOI:** 10.3390/plants13081079

**Published:** 2024-04-11

**Authors:** Tonka Ninčević Runjić, Marija Jug-Dujaković, Marko Runjić, Łukasz Łuczaj

**Affiliations:** 1 Institute for Adriatic Crops and Karst Reclamation, Put Duilova 11, 21000 Split, Croatia; masagatin@gmail.com (M.J.-D.); marko.runjic@krs.hr (M.R.); 2 Institute of Biology, University of Rzeszów, Ul. Zelwerowicza 4/451, Building D9, 35-601 Rzeszów, Poland; lukasz.luczaj@interia.pl

**Keywords:** ethnobotany, ethnomycology, wild food plants, Mediterranean diet

## Abstract

Background: Dalmatian Zagora has experienced significant depopulation trends over recent decades. The area is very interesting because of its rich biodiversity of species as well as its history of the use of wild foods. Since there is a danger of permanent loss of knowledge on the use of wild edibles, we focused our research on recording traditions local to this area. Methods: We conducted interviews with 180 residents. Results: A record was made of 136 species of wild food plants and 22 species of edible mushrooms gathered in the area. The most frequently collected species are *Rubus ulmifolius* Schott, *Cornus mas* L., *Portulaca oleracea* L., *Asparagus acutifolius* L., *Sonchus* spp., *Morus* spp., *Taraxacum* spp., *Amaranthus retroflexus* L., *Cichorium intybus* L., and *Dioscorea communis* (L.) Caddick & Wilkin. Conclusions: The list of taxa used is typical for other (sub-)Mediterranean parts of Croatia; however, more fungi species are used. The most important finding of the paper is probably the recording of *Legousia speculum-veneris* (L.) Chaix, a wild vegetable used in the area.

## 1. Introduction

The documentation of ethnobotanical knowledge holds significant scientific value, particularly in the present age, marked by rapid social transformations, diminishing plant biodiversity, and the erosion of traditional knowledge of wild plant uses [1]. 

In the Mediterranean part of Europe, there is a long tradition of using wild plants for nutrition and medicinal treatment. The Mediterranean diet is known worldwide to have many health benefits, and the consumption of wild Mediterranean foods has certainly played a part in this. The frequent collection of wild foods, especially wild green vegetables, can be seen as an important, but often overlooked, part of the Mediterranean diet [2,3]. There are numerous studies showing that the Mediterranean diet, rich in alpha-linolenic acid, is responsible for the prevention and suppression of cardiovascular disease [4,5]. Green vegetables, nuts, seeds, game, and wild seafood are good sources of omega-3 fatty acids, and wild-collected vegetables are particularly rich in antioxidants and alpha-linolenic acid [6,7].

The use of wild foods in southern Europe is unfortunately declining, but the memory of many interesting wild vegetables is still preserved [8,9,10,11,12,13,14,15,16,17]. In order to save this intangible cultural heritage from being lost, it is imperative that the last remnants of traditional knowledge and practices are captured, given their worrying decline [1]. 

Croatia is a Mediterranean country with rich plant biodiversity and a long tradition of using wild food and medicinal plants [14]. The first comprehensive ethnobotanical research in Croatia was carried out between 1962 and 1986 by Josip Bakić and his colleagues from the Institute of Naval Medicine of the Yugoslav Navy in Split [18]. Afterwards, there was a long pause in research on the use of wild plants in the area. This trend has changed, and new research has been carried out to fill this gap. Recent ethnobotanical studies have been carried out mainly in the Croatian coastal parts [12,14,19,20], and only to a lesser extent in the hinterland [21,22,23,24]. In a review article of ethnobotanical research in Croatia, authors Ninčević Runjić et al. [25] observed that rural inland areas remain scarcely investigated and are at the risk of permanent loss of traditional knowledge held by the local elder population. A look at the available literature shows that Dalmatian Zagora has not yet been sufficiently researched and represents a valuable source of traditional knowledge. In Dalmatian Zagora, only two smaller areas—Knin [23] and Poljica [22]—have been explored so far, with a large area remaining uninvestigated. Moreover, there is little data on the traditional use of edible mushrooms in Croatia, and research about it is very sparse [14,21,22,24].

Geographically, three natural borders and one state border define Dalmatian Zagora. The coastal mountain range is the border to the coastal part of Dalmatia, the river Krka is the border to the west, and Vrgorsko polje is to the east. In the north, the border overlaps with the state border of Bosnia and Herzegovina [26]. Large parts of Dalmatian Zagora have been included in the Natura 2000 ecological network. 

From a social perspective, Dalmatian Zagora has long been confronted with negative population growth due to depopulation, emigration, and aging of the population [27]. Historical upheavals and foreign invaders have had a strong influence on migration, but also on the customs of the inhabitants. In the late 15th and early 16th centuries, the Turks conquered most of Dalmatian Zagora. For the next hundred years, this area was the border between the Ottoman and Christian worlds [26]. Emigration processes in Dalmatian Zagora began a long time ago and have intensified in the second half of the 20th century through the process of littoralization and deruralization, meaning that economic activities, populations, and settlements become increasingly concentrated in coastal regions. This trend often involves the abandonment of inland settlements, leading to a migration of both people and resources from the interior towards the coast [28]. The influence of littoralization on Dalmatian Zagora can be seen in the processes of urbanization, deruralization, industrialization, and tertiarization. At the same time, this area has remained ecologically preserved due to the lack of hard industry [29]. All of the above factors, as well as changing dietary habits, have contributed to the decline of traditional knowledge and practices related to wild food gathering and indicate the urgent need to document remaining knowledge. Thus, the aim of the study was to record the use of wild plants and fungi as food and in drinks in Dalmatian Zagora. The findings of this study will contribute to our understanding of the botanical richness within the region and underscore the importance of preserving traditional knowledge before it is lost to time.

## 2. Results

We recorded 136 species of edible plants used in the area (Table 1). On average, a respondent mentioned 15 species of wild food plants. The most cited edible plant species were *Rubus ulmifolius* Schott, *Cornus mas* L., *Portulaca oleracea* L., *Asparagus acutifolius* L., *Sonchus* spp., *Morus* spp., *Taraxacum* spp., *Amaranthus retroflexus* L., *Cichorium intybus* L., and *Dioscorea communis* (L.) Caddick & Wilkin.

We also recorded 22 taxa of edible fungi (Table 1), but only 16 taxa are eaten by more than one informant. On average, 0.8 species of fungi were mentioned per interview (only 30 informants, i.e., 18% of them, mentioned gathering fungi). The most commonly mentioned were *Agaricus* spp., *Boletus edulis*, *Lactarius* section *Deliciosi*, *Macrolepiota*, and *Cantharellus cibarius*. Fungi are gathered mainly in Sinj and Vrlika, with reports from seven informants per settlement out of the total 30 for the whole studied area. 

In the entire Dalmatian Zagora, the largest number of taxa in terms of the mean number of species listed per interview was recorded in the Sinj area (25 species), then followed by Zagvozd and Podbablje (22) and Runović and Zmijavci (20). The lowest mean number of species was recorded in Šestanovac, Zadvarje, and Omiš (10), Prgomet (12), and Unešić and Ružić (12). There was a moderate positive correlation (*r* = 0.51, *p* = 0.044) between the mean number of species mentioned per interview and the number of inhabitants in each of the 16 studied units (regions). The units in which more than the average numbers of species were mentioned tend to be located east of Split, and those with the lowest knowledge were in the west. The number of cited species exceeded forty in four interviews: two from Sinj (sixty-one and forty-seven species), one from Podbablje (forty-five species), and another from Runovići (forty-four species).

The most collected parts were green parts (47%), followed by fruits (41%), flowers and flowering shoots with leaves (9%), and underground parts (3%).

Mišanca, also called divlje zelje, is the most commonly made wild dish, prepared in all parts of Dalmatian Zagora from different wild vegetables such as *Sonchus oleraceus*, *Taraxacum officinale*, *Cichorium intybus*, *Allium ampeloprasum*, *Chenopodium album*, *Bunias erucago*, *Viola tricolor*, *Rumex* sp., *Foeniculum vulgare*, *Allium vineale*, *Bunias erucago*, *Silene vulgaris*, *Papaver rhoeas*, and *Capsela bursa-pastoris.* Vegetables are boiled for a short time, often with the addition of potatoes. At the end, they are seasoned with salt and olive or sunflower oil. 

The second most common dishes mentioned by respondents were *Dioscorea communis* and *Asparagus officinalis* eaten as a raw salad, or briefly cooked or fried with eggs, and seasoned with olive oil or vinegar. *Portulaca oleracea* was often mentioned as a favorite single-species salad. 

A specialty associated with the Imotski region is the plant grzdulja, grdulja, i.e., *Bunias erucago*. It is mentioned numerous times by informants and also occurs in the records of the priest Silvestar Kutleša (1876–1943) [30], who wrote down the folk knowledge and customs of the people in the Imotski region. In the book, he mentions the frequent folk use of other wild plants: *šurlin* (*Capsela bursa-pastoris*), *sparoga* (*Asparagus officinalis*), *koprva* (*Urtica* sp.), and *bljušt* (*Dioscorea communis*). *Grzdulja* used to be boiled and seasoned with oil, butter, or lard; today, it is usually cooked with dried meat or as a vegetable pie. 

Mushrooms are usually fried and served as a side dish and rarely cooked in stew (goulash or sauce). Apart from the drying of *Boletus edulis*, there is no tradition of mushroom preservation. The use of mushrooms in villages near Trilj and Sinj is related to their location by the river Cetina, where the agroclimatic conditions are suitable for their growth.

Wild fruits are often mentioned as having versatile uses. They are usually used ripe for immediate consumption or, more rarely, dried. They are also made into jam, liqueur, or compote. One frequently mentioned species is *Rubus ulmifolius* Schott. Older interviewees, in particular, stated that they ate it as children but also picked it for sale and used the money earned to buy books for school. *Celtis australis*, *Cornus mas*, *Prunus spinosa*, *Morus nigra*, and *M. alba* were mostly eaten while herding cattle on the pastures. The importance of the mulberry tree for the population’s diet is demonstrated by the popular saying “Pure i murve”, “polenta and mulberry”, which were eaten together as a poor man’s meal. *Aria edulis*, *Torminalis glaberrima*, *Sorbus domestica*, *Pyrus communis subsp. communis*, and *Malus sylvestris* were frequently eaten in the past, but respondents state that they rarely consume them today.

Some of the plants mentioned in the list are only used to flavor traditional alcoholic drinks, mainly *travarica*, where the >40% rakija contains a mix of several aromatic herbs, most commonly *Satureja*, *Salvia rosmarinus*, *Laurus nobilis*, *Foeniculum vulgare*, *Teucrium* sp., etc. Two respondents also mentioned a new trend of making *jeger*, a homemade Jägermeister-like drink inspired by the famous German liqueur. The difference between *travarica* and *jeger* is that the latter is made with plants more typical of the continental part of Croatia, as the people have borrowed the recipe from Germany; it is also sweetened, in contrast to the dry *travarica* made with aromatic Mediterranean herbs.

## 3. Discussion

The number and composition of wild foods gathered in the area is similar to other parts of southern Croatia [14,19,21,22]. In the research conducted in Dubrovnik, the usage of 95 wild edible species was documented [14]. On the Adriatic islands, 89 taxa of wild vegetables were identified [19], whereas 55 species were recorded in the Zadar area [21]. Additionally, the number of wild foods in the Krk and Poljica regions was 80 and 76, respectively [22]. The composition of wild foods comprising mainly wild vegetables is typical for the Mediterranean in contrast to Central Europe where the use of fruits and mushrooms dominates [11,31]. 

Although the most commonly mentioned species are widely known as edible, we found at least the memory of the use of some more unusual food plants. 

*Legousia speculum-veneris* (L.) Chaix was mentioned by several informants as a wild vegetable eaten in the area. The use of this species as food has been reported in the literature only by Paoletti [32] in the Friuli region in NE Italy.

Another interesting species is *Arum italicum* Mill. Although plants from this genus are occasionally used in southwestern Asia and the Caucasus [33,34,35,36], it is not widely used in Europe nowadays as a food plant due to it incredibly sharp taste when eaten raw or underprocessed, owing to the presence of oxalates. According to Paura and Di Marzio [37], Arum sp. has been utilized as a food source across various regions of Europe, particularly valued for the starch extracted from its tubers, which is used in bread preparation. In Bosnia, the tubers of *Arum italicum* and *A. maculatum* continue to be employed in the cooking of boiled meats or focaccia [38]. Albania had a history of consuming *Arum italicum* during times of scarcity, with its usage evolving over time [39]. Additionally, the leaves are consumed in southeastern Europe after being boiled repeatedly, while in Switzerland Arum leaves are ingested in spring as part of a cleansing regimen [40]. In Italy, *Arum italicum* and *A. maculatum* are predominantly recognized for their medicinal properties, finding application in various ailments [41].

One more interesting species is *Centaurea solstitialis* L., previously mentioned as used only in the Ravni Kotari area near Vrana [21]. In the abovementioned paper, the species was only reported with the local name kravlja gubica (the same as in Zagora, literally “cow’s face”) and it was only a year after the publication of the paper that the authors identified the taxon, which is also eaten in Turkey [42].

It should be noted that over a third of the species in this study were mentioned only by one informant. The researcher is in a difficult position when assessing the use of species mentioned only by a single informant, as this information may be confounded by several factors:

1. Relic uses—species once used more frequently, now remembered by one person;
2. Rare uses—in cases when the plant was never an important useful species used only, e.g., as famine food;
3. Idiosyncratic uses—uses restricted to one person are sometimes developed by them through experimentation or observation of foraging animals, e.g., sheep, goats, or pigs;
4. Mistaken identification—when the informant does not remember the species well or supplies a mistaken voucher specimen.

In the case of our study, in most instances, we could assess the reliability of one-informant mentions by comparing local names and uses of plants in the neighboring regions. Some doubts remained concerning two uses not recorded elsewhere in the Balkans: *Fumaria officinalis* and *Erythronium dens-canis. F. officinalis* is a relatively toxic medicinal plant, so it was surprising to see it in a wild vegetable mix, and care should be taken with using it. In the case of *E. dens-canis*, ours is the first record of its consumption in the Balkans. The species has edible, starchy, and delicious underground corms. Only Sturtevant [43] mentions the use of the species in west or central Asia, quoting Gmelin (1747) [44]. He wrote that Tartars collected and dried the bulbs and boiled them with milk or broth [43], but this probably refers to the species sensu lato, now classified as *E. sibiricum.* The latter was widely eaten in Siberia [45], and *E. japonium* was consumed by the Ainu in Japan [46]. The American species of *Erythronium* were an important food item for the ethnic groups that lived in the western part of North America [47].

The presented data show that wild edible mushrooms are gathered by many inhabitants, although the inventory of most frequently used species is not long. By comparing out findings with other data on fungi uses in Europe, we could conclude that the local population is somewhere in the middle of the mycophilia–mycophobia spectrum invented by Wasson [48]. Some species are known by a portion of the population but there are also people who neither know or collect them. The reason for the relatively low interest in mushroom gathering may be the dry climate and the scarcity of wooded areas near settlements.

The relatively large list of wild vegetables and the frequent use of several of them place the local population high on the herbophilia spectrum [31], typically for the Mediterranean part of Europe, where gathering wild vegetables is one of the important though overlooked parts of the Mediterranean diet [2,3,13,49,50,51,52,53,54].

Another use of wild taxa recorded in research area was for preparation of traditional alcoholic beverages, mainly *travarica* (grape pomace distillate flavored with single or mixed species), that have received limited attention from researchers so far in Croatia [24,55]. What is worth highlighting is that nearly all the plants mentioned are either wild or cultivated locally, and the alcohol is produced locally, showing that traditional alcoholic beverages have a great role in the traditional culture and social life of the studied communities. This particularity related to alcoholic beverages was also recorded in the Tuscany and Emilia-Romagna regions [56,57].

Recently Łuczaj [1] wrote about the urgent need to record the disappearing uses of plants in the world. The large number of uses mentioned only by single individuals in this study illustrates the devolution of human–plant interactions. Here, in Dalmatian Zagora, some uses that were probably widely known by most inhabitants of many villages are now remembered by a single person in one.

## 4. Materials and Methods

### 4.1. Study Area

**Figure 1:**
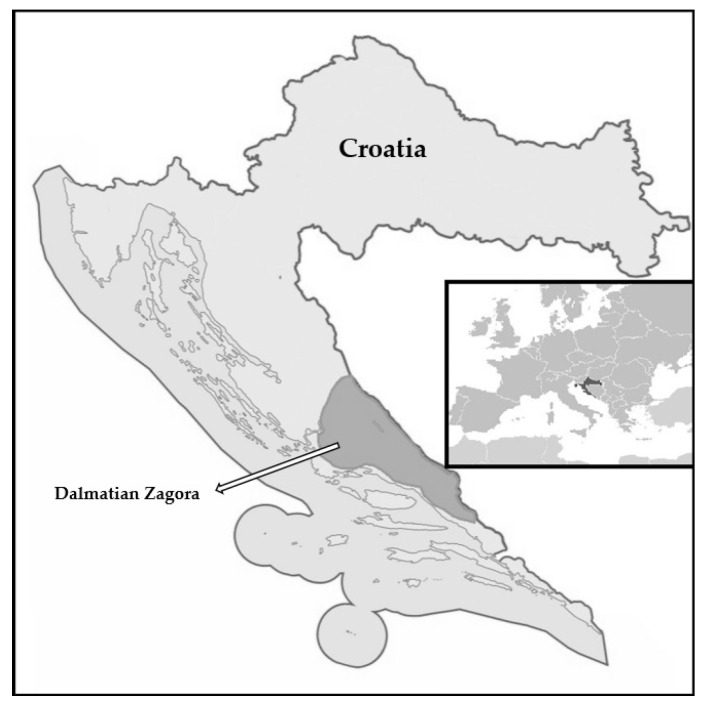
The area of Dalmatian Zagora.

Dalmatian Zagora, while not an officially recognized administrative unit, is a conceptually expansive term delineating a region outlined by Delić [58]. Dalmatian Zagora represents part of the Dalmatian hinterland, and exact boundaries are given by the following authors. The definition by Faričić and Matas [26], corroborated through empirical research by Vukosav [28,59], forms the basis of its geographical extent, encompassing areas beyond the coastal hills of Trtar (738 m), Opor (650 m), Kozjak (780 m), Mosor (1330 m), Omiška Dinara (864 m), Biokovo (1762 m), and Rilić (1155 m). Its northern border aligns with the state border of Bosnia and Herzegovina, the western frontier is traced by the Krka basin, and the eastern border includes Vrgorsko polje and Rastok polje (Figure 1).

Administratively, Dalmatian Zagora spans the territory of two counties: Split-Dalmatia and Šibenik-Knin, comprising 8 cities, 25 municipalities, and 293 settlements [60]. The 2021 Census reported 157.534 inhabitants within Dalmatian Zagora, among which there were 90.282 rural residents [61]. Dalmatian Zagora included a quarter of the population of the two counties in which it is located. At the same time, Dalmatian Zagora covered over 50% of the territory of these two counties. This disparity in population density is a consequence of littoralization and exceptional polarization between the coast and the hinterland [58]. Dalmatian Zagora sustains an average population density of 31 inhabitants/km^2^, notably lower than the Republic of Croatia’s 76 inhabitants/km^2^. As a result of numerous historical upheavals, this region witnessed a population decline of 81,011 inhabitants in four decades, signifying a reduction of over a third from its initial population [58].

Climate categorization, according to the Köppen classification, reveals two prevailing types in Dalmatian Zagora: a moderately warm humid climate with hot summers (Cfa) and a moderately warm humid climate with warm summers (Cfb) [62]. The region’s climate exhibits sub-Mediterranean characteristics closer to the coast, gradually transitioning to stronger continental influences farther inland. Vegetation primarily comprises degraded forms of maquis and garrigue [63].

Geographically, Dalmatian Zagora is characterized by limited arable land, with only single larger karst fields. Consequently, developmental opportunities in this region remain constrained, with agricultural pursuits—specifically arable farming and extensive animal husbandry—forming the primary economic activities [64].

### 4.2. Field Study

In this study, we included the entire Dalmatian Zagora, except the Poljica and Knin area, as it has already been comprehensively studied by Dolina et al. [22] and Varga et al. [23]. Initially, our field research plan aimed for a minimum of 15 interviews per local unit. However, upon visiting the settlements, we encountered challenges in identifying knowledgeable informants, owing to the region’s sparse population density. For this reason, we had to merge certain settlements into one unit, resulting in a total of 16 units instead of 19 (Table 2). 

The population of Dalmatian Zagora is gathered mostly around a few centers that have formed smaller historical regions: Drniš region, Imotski region, Knin region, Omiška (Poljička) Zagora, Sinj (Cetina) region, Vrgorac region, and Zagora (in the narrower sense) [26].

We conducted semi-structured interviews from March 2021 to September 2023. Data were collected mainly using the free listing method. The informants were selected following the snowball method [65] or were encountered during their work in the fields. Interviews were conducted in Croatian. The criterion was to examine only local residents, or those who have lived in the area for most of their lives. When possible, we organized walks with selected key informants to show us precisely which plants they collected. Plants were mostly identified on-site; otherwise, specimens were collected and identified by an experienced botanist (see Acknowledgments) using standard identification keys and iconographies [66,67].

We collected information about the respondents’ age, gender, place of residence, and place of origin. Altogether, 170 interviews were conducted with 195 people (145 interviews with single respondents and 25 interviews with 2 respondents). Among the respondents, 115 were women (59%) and 80 were men. The age of the interviewees ranged from 31 to 95 (average age = 66.38 median = 66). 

Interviewees were asked questions about collected wild vegetables, roots, fruits, and mushrooms; their preparation methods; and the plant parts utilized. This study was conducted following the guidelines of the International Society of Ethnobiology Code of Ethics [68] and the American Anthropological Association Code of Ethics [69]. Voucher plant specimens were collected and deposited in the Herbarium Croaticum (ZA) at the University of Zagreb, Faculty of Science. Voucher fungi specimens were collected and deposited at the Department of Plant Sciences, Institute for Adriatic Crops and Karst Reclamation. Plant nomenclature adhered to Plants of the World Online [70]. 

The data matrix was stored in Microsoft Office 365 Excel version 2402. Correlations were calculated using the same program with Pearson’s correlation coefficient [71].

## 5. Conclusions 

The studied region exhibits a rich diversity of edible plants indicating a significant knowledge of wild food resources. The wild plants consumed in the region studied are typical of this part of the Mediterranean and differ most from the Croatian coastal area in the higher consumption of mushrooms. There were some regional differences in the knowledge of edible plants and a moderate positive correlation was observed between the mean number of species mentioned per interview and the number of inhabitants in each region. Traditional dishes like “Mišanca”, made from various wild vegetables, are being prepared throughout the researched area, and specialty dishes containing *Bunias erucago* are specific only to certain regions within the area. Many plants are used to flavor traditional alcoholic drinks, showing a connection between local flora and cultural practices. The memory of the use of some other unusual food plants was also recorded: *Legousia speculum-veneris*, *Arum italicum*, and *Centaurea solstitialis.* The study was probably the last chance to document the fading tradition of wild food plant usage in this part of Croatia. The results will contribute to the general understanding of ethnobotany of the Mediterranean. 

## Figures and Tables

**Table 1:** Wild taxa of fungi and plants consumed in the study area.

Scientific Name	Local Name (s)	FB—Fruiting Body, FR—Fruit, L—Leaves, R—Underground Part, WH—Whole above Ground Part (Flowers and Leaves)	Preparation Method	Number of Use Reports	Voucher Specimen Number
Fungi					
*Agaricus* cf *macrosporus* (F.H.Møller & Jul.Schff.) Pilát	kračun	FB	fried	7	WA01
*Agaricus* spp. (other smaller species)	šampinjon, pečurka	FB	cooked, fried	29	WA02
*Amanita caesarea* Scop.	blagva	FB	fried, raw	9	WA03
*Armillaria* sp.	puza	FB	boiled	1	
*Boletus edulis* Bull.	vrganj	FB	boiled	16	WA04
*Cantharellus cibarius* Fr.	lisičarka	FB	boiled	9	WA05
*Chroogomphus rutilus* (Schaeff.) O.K.Mill.	borov čavlić	FB	boiled	2	
*Clitocybe nuda* (Bull.) H.E.Bigelow & A.H.Sm.	modrikača	FB	boiled	1	
*Craterellus cornucopioides* (L.) Pers.	trubača	FB	boiled	1	WA06
*Coprinus comatus* (O.F.Müll.) Pers.	gnjojištarka, gnojištarke	FB	boiled	2	
*Hydnum repandum* L.	prosenjak	FB	boiled	2	WA07
*Hygrophorus russula* (Schaeff.) Kauffman	medenka	FB	boiled	1	
*Imleria badia* (Fr.) Vizzini	kostanjevka	FB	boiled	1	WA08
*Infundibulicybe gibba* (Pers.) Harmaja	martinčica	FB	boiled, fried	5	WA09
*Lactarius* sect. *Deliciosi*	rujnica	FB	boiled	13	WA010
*Leccinum aurantiacum* (Bull.) Gray or *Suillus* sp.?	osinac	FB	boiled	1	
*Lycoperdaceae*	puhara	FB	boiled	2	WA011
*Macrolepiota* sp.	sunčanica	FB	boiled, fried	14	WA012
*Morchella s*p.	smrčak, smrčka	FB	boiled	6	WA013
*Pleurotus ostreatus* (Jacq. ex Fr.) P.Kumm.	bukovača	FB	boiled	2	WA014
*Ramaria* sp.	koraljka	FB	pickled	1	
*Suillus granulatus* (L.) Vill.	slinavac, krušćić, slinavka	FB	boiled	4	
Plants					
*Achillea millefolium* L.	stolisnik, hajdučka trava	WH	infusion, travarica	14	78776
*Alchemilla* sp.	divlja vrkuta	L	jeger	1	78854
*Alliaria petiolata* (M. Bieb.) Cavara & Grande	češnjača	L	boiled	1	78804
*Allium ampeloprasum* L.	divlji luk, divji luk	WH	boiled	24	78824
*Allium* sp.	divlji luk, divji luk	WH	boiled	28	78780
*Allium ursinum* L.	medvjeđi luk, divlji luk, sličke	WH	boiled	5	78855
*Allium sphaerocephalon* L.	koziroga	WH	boiled	1	78856
*Allium nigrum* L.	žbun-luk	WH	boiled	2	78857
*Allium vineale* L.	jukelj, ljukelj	WH	boiled	15	78858
*Allium paniculatum* L.	balučka	WH	boiled	2	78859
*Amaranthus retroflexus* L.	štir, šćir	L	raw snack, boiled	79	78860
*Anchusa officinalis* L.	boražina	WH	raw snack	2	78863
*Arbutus unedo* L.	maginja	FR	raw snack, liqueur	2	78765
*Arctium* sp.	čičak	L	mišanca	2	78861
*Aria edulis* (Willd.) M.Roem. (syn. *Sorbus aria* (L.) Crantz)	mukinja	FR	raw snack	15	78782
*Artemisia absinthium* L.	pelin	WH	travarica, liqueur	9	78807
*Arum italicum* Mill.	kozlac	L	mišanca	4	78799
*Asparagus acutifolius* L.	šparoga	SH	raw snack, boiled	99	78764
*Asphodelus* sp.	sindulji	R	raw snack	2	78823
*Bellis perennis* L.	tratinčica	WH	mišanca	3	78826
*Beta vulgaris* L.	blitva	L	mišanca, jeger	4	78862
*Bunias erucago* L.	grzdulja	L	boiled on its own or in mišanca	33	78864
*Capsela bursa-pastoris* (L.) Medik.	šurlin, šurin, rusomača	L	mišanca	23	78788
*Castanea sativa* L.	kesten	FR	jeger		78865
*Caucalis platycarpos* L.	podlanica	L	mišanca	1	78793
*Celtis australis* L.	kostela, koštela, kostelja, kostila, košten	FR	raw snack, liqueur, jam	64	78866
*Centaurea solstistalis* L.	kravlja gubica		boiled	1	78867
*Centaurium* sp.	kičica	WH	travarica	1	78868
*Chenopodium album* L.	loboda	L	mišanca	58	78811
*Chondrilla juncea* L.	mličika	L	mišanca	4	78869
*Cichorium intybus* L.	žutinica, konjogriz, radić, žuća, cikorija, jastrebljak, žutenica, ženetriga, vodopija, mličika	L, R	boiled, raw snack, dried and roasted for beverage	76	78870
*Clinopodium nepeta* (L.) Kuntze	[no name given]	WH	travarica	2	78871
*Colutea arborescens* L.	pucaljke	L, FR	young shoots and seeds, raw as children’s snack	1	78872
*Convolvulus arvensis* L.	slak, slavak	L	mišanca	1	78873
*Cornus mas* L.	drijenak, drijen, drinja, drinjina, drina, drin, drinak, dren, drinovina, drenjina	FR	cooked for jam; in grappa, juice, liqueur, and travarica	112	78834
*Crataegus monogyna* Jacq.	glog, gloginja, bili trn	FR	raw snack, travarica	58	78874
*Crepis sancta* (L.) Bornm.	zečevac	L	mišanca	1	78786
*Crepis* sp.	krepis	L	mišanca	1	78832
*Daucus carota* L.	divlja mrkva, iglica, lovačka mrkva	FR, L, R	boiled, raw snack	32	78875
*Dioscorea communis* (L.) Caddick & Wilkin	kuka, blješt, blušć, kukača, bljušt, kukenjač	SH	boiled, raw snack	74	78822
*Diplotaxis tenuifolia* (L.) DC. and *D. erucoides* (L.) DC.	riga, divlja riga, nadimača, divlja rikula	L	mišanca, raw snack	7	78815
*Erodium cicutarium* (L.) L’Hér.	iglica, čapljan	L	mišanca	3	78827
*Eryngium amethystinum* L.	brnbeč	L	mišanca	1	78876
*Erythronium dens-canis* L.	pasji zub	R	boiled, raw snack	1	78877
*Ficus carica* L.	smokva	FR, L	juice, liqueur (FR), infusion (L)	2	78879
*Filipendula vulgaris* Moench	končara	R	raw snack	1	78880
*Foeniculum vulgare* Mill.	koromač, komorač	L, FR, stem	raw snack, mišanca, travarica, spice in sausage	71	78810
*Fragaria vesca* L.	šumska jagoda, medina jagoda, divlja jagoda	FR	mostly raw snack (FR), infusion (L)	21	78789
*Fraxinus ornus* L.	crni jasen	sap	sap	1	78881
*Fumaria* cf. *officinalis* L.	dimnjača	L	mišanca	1	78796
*Galium aparine* L.	broč, ljepljiva bročika	L	travarica	1	78798
*Gentiana lutea* L.	srčanik	R	travarica	7	78882
*Geranium lucidum* L.	krvavac	L	mišanca	1	78802
*Helianthus tuberosus* L.	divlji krumpir, čičoka, artičoka	R	boiled	1	78883
*Helichrysum italicum* (Roth) G.Don	smilje	WH	travarica	1	78766
*Heracleum sphondylium* L.	medvjeđa šapa, vučja šapa	L	mišanca	5	78884
*Humulus lupulus* L.	hmelj	SH	boiled like asparagus	1	78885
*Hypericum perforatum* L.	gospina trava, kantarion	WH	travarica	4	78781
*Juglans regia* L.	orah	FR	liqueur	22	78886
*Juniperus communis* L.	borovica, kleka	FR, L	travarica	4	78887
*Juniperus macrocarpa* Sm.	pukinja, pukinjaš	FR	travarica, raw	3	78888
*Juniperus oxycedrus* L.	smrič, smrikovina	FR	travarica, raw	26	78771
*Lactuca serriola* L.	divlja salata, salatuša	L	mišanca	4	78791
*Lamium* sp.	crljenak	L	mišanca	1	78829
*Laurus nobilis* L.	lovor	L	culinary herb for food, travarica	22	78767
*Legousia speculum-veneris* (L.) Chaix	zečje mudance, venerina zrcalica, mišje mudance, mačje mudance	L	mišanca	7	78889
*Leucanthemum vulgare* Lam.	ivančica	L	mišanca	1	78890
*Malus* sp.	divlja jabuka, divljakinja	FR	raw snack, boiled, vinegar	27	78893
*Malva sylvestris* L.	sljez	L	mišanca	3	78812
*Melissa officinalis* L.	matičnjak	WH	travarica	2	78891
*Mentha* spp. (*M. x piperita* L. & *M. spicata* L.)	menta	L	infusion, juice, culinary herb, travarica	11	78787
*Morus alba* L. & *M. nigra* L.	bijela murva, bila, crna, crvena murva, dud žutica i crnica (VC)	FR	raw snack, syrup, liqueur	95	78894, 78895
*Myrtus communis* L.	mirta	FR	travarica	1	78892
*Nigella sativa* L.	crni kim, žuta žila	L	mišanca	2	78896
*Origanum vulgare* L.	origano, mravinac	WH	culinary herb	14	78773
*Paliurus spina-christi* Mill.	drača (the fruit is called ‘šeširić’)	FR	travarica	3	78897
*Papaver rhoeas* L.	mak, makalj, kukurik	L	mišanca	72	78817
*Parietaria judaica* L.	crkvina	L	mišanca	2	78774
*Plantago lanceolata* L. (mainly) & *P. major* L.	trputac	L	mišanca	13	78830
*Portulaca oleracea* L.	tušt	WH	raw snack, mišanca	106	78898
*Primula vulgaris* Huds.	jaglac, jagorčevina	WH	raw snack, jeger	3	78899
*Prunus cerasifera* Ehrh.	divlja šljiva, đenerika, zerdelija, zerzelija, srdelija, razdelija, zerdelinka, šlama, srndelija, drndelija, zerzelinka, vinika, vinka	FR	raw snack, jam, juice	50	78900
*Prunus mahaleb* L.	rašeljka, rašejka	FR	liqueur, jam, raw snack	6	78901
*Prunus persica* L.	vinogradska breskva	FR	raw	1	78902
*Prunus spinosa* L.	trnina, trn sv. Ante, crni trn, gloginje	FR	raw snack, liqueur, jam	58	78903
*Punica granatum* L.	šipak, ljutur, ljuti šipak	FR	syrup, jam	3	78904
*Pyrus communis* L. subsp. *communis*	divlja kruška, divja kruškica	FR	raw snack	29	78835
*Pyrus spinosa* Forssk.	krušvina, divlja kruška, divja kruška	FR	raw snack	25	78905
*Quercus* sp.	žir, hrast	FR	roasted as coffee substitute	4	78769
*Ranunculus* sp.	vučja stopa, vučja stopica, vrania nožica	L	boiled	14	78831
*Rhagadiolus stellatus* (L.) Gaertn.	bila mličika, kosovac	L	mišanca	1	78878
*Robinia pseudoacacia* L.	bagrem	FL	boiled	1	78906
*Rosa canina* L.	šipurika, divlji šipak, svrbiguzica, šepurina, svrbiguza	FR	infusion, jam, liqueur	22	78907
*Rosa x centifolia* L.	ruža	FL	liqueur, syrup	7	
*Rubus idaeus* L.	malina	L	infusion	5	78908
*Rubus ulmifolius* Schott	kupina, jagoda	FR	raw snack, syrup, jam, liqueur, wine (FR), infusion (L)	122	78833
*Rumex* spp. (*R. pulcher* L., *R. crispus* L. and *R. acetosa* L.)	štavelj, štavolj, šćavelj, štavalj, ljuta trava, kiselica, kiseljača, teta lija	L	mišanca, raw snack	52	78800 78816 78792
*Ruscus aculeatus* L.	marine jagode, koprčine (Radošić)	SH	boiled	1	78909
*Ruta graveolens* L.	rutica, rutva	FL, L	travarica	3	78910
*Salvia officinalis* L.	kadulja	L	syrup, liqueur, travarica, culinary herb	36	78778
*Salvia rosmarinus* Spenn. (syn. *Rosmarinus officinalis* L.)	ružmarin, ruzmarin	L	culinary herb, travarica	40	78775
*Sambucus nigra* L.	bazga, zova, zovina, zovkovina	FL, FR	syrup, liqueur, jam, boiled	47	78911
*Sanguisorba minor* Scop. subsp. *balearica* (Nyman) Muñoz Garm. & C. Navarro	krvara, mala krvara	L	mišanca	2	78801
*Satureja montana* L.	vrisak	L	culinary herb, travarica	24	78777
*Scandix pecten-veneris* L.	venerin češalj, koromačika	L	mišanca	1	78814
*Scorzonera* sp. & *Gelasia villosa* (Scop.) Cass.	turutva, turita, kozja brada	L, R	mišanca	21	78819
*Silene latifolia* Poir.	volujsko uho, volovo, volunje uho, zečje uši, ušac, volunjski ušac, tušac	L	mišanca	16	78794
*Silene vulgaris* (Moench) Garcke	škripavac, pucavac, pušina, pucalina	L	mišanca	26	78808, 78797
*Smyrnium* cf *perfoliatum* L.	prorasla lesandra, lesandra	L, T	boiled	2	78912
*Sonchus* spp. (*S. oleraceus* L. and *S. asper* (L.) Hill.)	kostrić, kostriš, radić, trnak, bijeli trnak, trnjak, brnbeć, skulovac (Prim. Dolac), kozja brada, šušak	L	mišanca	92	78805
*Sorbus domestica* L.	oskoruša	FR	raw snack, liqueur, jam	35	78770
*Torminalis glaberrima* (Gand.) Sennikov & Kurtto (syn. *Sorbus torminalis* (L.) Crantz)	brekinja	FR	raw snack	9	78768
*Stachys cretica* L.	ranjenik	L	mišanca	1	78913
*Stellaria media* (L.) Vill.	mišjakinja	L	mišanca	1	78914
*Taraxacum* spp.	maslačak, divlji radič, radić, smrčika, slačak, mličika	L	mišanca	75	78821
*Teucrium chamaedrys* L.	dubčac	WH	travarica	1	78820
*Teucrium montanum* L.	trava iva	WH	travarica	11	78806
*Thymus longicaulis* C. Presl & *T. serpyllum* L.	majčina dušica	WH	culinary herb, travarica	14	78809 78772
*Tilia* sp.	lipa	FL	infusion	1	78915
*Tordylium apulum* L.	mačja muda	L	mišanca	1	78916
*Tragopogon* sp.	kozja brada	L	mišanca	5	78818
*Tussilago farfara* L.	podbijel	WH	jeger	1	78917
*Urtica dioica* L. and other *Urtica* spp.	koprva, kopriva, žara	L	boiled	63	78828
*Valerianella* cf. *locusta* L.	lucina pica, divlji matovilac, macina pica	L	mišanca	12	78813
*Veronica persica* Poir.	čestoslavica	L	mišanca	1	78795
*Vicia faba* L.	divlji bob	L	mišanca	1	78803
*Viola odorata* L.	ljubičica	FL	raw snack, jeger	4	78779
*Viola arvensis* Murray	kokošija volja, kokošja volja, kokošija voljica, kokina voljica, šena	WH	mišanca	32	78783

**Table 2:** List of the studied units (municipalities).

Unit No.	Municipalities and Towns	Number of Species per Interview	Rural Population *	Settlements
1	Vrgorac	16	3566	Banja, Dragljane, Draževitić, Duge Njive, Dusina, Kljenak, Kokorići, Kotezi, Kozica, Mijaca, Orah, Podprolog, Poljica, Kozička, Prapatnice, Rašćane, Ravča, Stilja, Umčani, Veliki Prolog, Vina, Višnjica, Vlaka, and Zavojane
2	Runović	20	1968	Podosoje, Runović, and Slivno
	Zmijavci		1654	Zmijavci
3	Zagvozd	22	957	Biokovsko Selo, Krstatice, Rastovac, Rašćane Gornje, Zagvozd, Župa, and Župa Srednja
	Podbablje		4035	Drum, Grubine, Hršćevani, Ivanbegovina, Kamenmost, Krivodol, Podbablje Gornje, and Poljica
4	Imotski	15	5145	Donji Vinjani, Glavina Donja, Glavina Gornja, Gornji Vinjani, Imotski, and Medvidovića Draga
	Proložac		3112	Donji Proložac, Gornji Proložac, Postranje, Ričice, and Šumet
	Lokvičići		667	Dolića Draga and Lokvičići
5	Lovreć	15	1402	Dobrinče, Lovreć, Medovdolac, Opanci, and Studenci
	Cista Provo		1799	Aržano, Biorine, Cista Velika, Cista Provo, Dobranje, and Svib
6	Šestanovac	10	1699	Grabovac, Katuni, Kreševo, Šestanovac, and Žeževica
	Zadvarje		289	Dupci, Kraljevac, Krnići, Krželji, Pejkovići, Potpoletnica, Santrići, and Zadvarje
	Omiš		6020	Nova Sela, Podašpilje, Slime, Svinišće, Kučiće, Blato na Cetini, Čisla, Donji Dolac, Dubrava, Gata, Gornji Dolac, Kostanje, Ostravica, Podgrađe, Putišići, Seoca, Srijane, Svinišće, Trnbusi, Tugare, and Zvečanje
7	Trilj	16	8182	Bisko, Budimir, Čačvina, Čaporice, Gardun, Grab, Jabuka, Kamensko, Košute, Krivodol, Ljut, Nova Sela, Podi, Rože, Strizirep, Strmrndolac, Tijarica, Trilj, Ugljane, Vedrine, Velić, Vinine, Vojnić Sinjski Voštane, Vrabač, and Vrpolje
8	Otok	15	4998	Gala, Korita, Otok, Ovrlje, Ruda, and Udovičić
9	Dugopolje	13	3742	Dugopolje, Koprivno, Kotlenice, and Liska
	Dicmo		2805	Dicmo, Ercegovci, Kraj, Krušvar, Osoje, Prisoje, Sičane, and Sušci
10	Sinj	25	12,681	Bajagić, Brnaze, Čitluk, Glavice, Gljev, Jasensko, Karakašica, Lučane, Obrovac Sinjski, Radošić, Sinj, Suhač, Turjaci, and Zelovo
11	Hrvace	14	3144	Dabar, Donji Bitelić, Gornji Bitelić, Hrvace, Laktac, Maljkovo, Potravlje, Rumin, Satrić, Vučipolje, and Zasiok
12	Vrlika	15	1728	Garjak, Ježević, Koljane, Kosore, Maovice, Otišić, Podosoje, and Vinalić i Vrlika
	Civljane		171	Cetina and Civljane
13	Muć	13	3465	Bračević, Crivac, Donje Ogorje, Donje Postinje, Donji Muć, Gizdavac, Gornje Postinje, Gornje Ogorje, Gornji Muć, Mala Milešina, Neorić, Pribude Radunić, Ramljane, Sutina, Velika Milešina, and Zelovo Sutinsko
	Klis		1730	Brštanovo, Dugobabe, Konjsko, Korušce, Nisko, Prugovo, Veliki Bročanac, and Vučevica
	Lećevica		495	Divojevići, Kladnjice, Lećevica, and Radošić
14	Prgomet	12	498	Bogdanovići, Labin, Prgomet, Sitno, and Trolokve
	Primorski Dolac		686	Primorski Dolac
	Unešić		1269	Cera, Čvrljevo, Donje Planjane, Donje Utore, Donje Vinovo, Gornje Planjane, Gornje Utore, Gornje Vinovo, Koprno, Ljubostinje, Mirlović Zagora, Nevest, Ostrogašica, Podumci, Unešić, and Visoka
15	Ružić	12	1283	Ružić, Gradac, Otavice, Moseć, Kljaci, Baljci, Umljanovići, Čavoglave, and Mirlović polje
16	Drniš	19	3524	Žitnić, Pokrovnik, Pakova selo, Radonić, Siverić, Trbounje, Velušić, Badanj, Bogatić, Brištane, Drinovce, Kadinu Glavicu, Kanjane, Kaočine, Karalić, Ključ, Kričke, Lišnjak, Parčić, Sedramić, Širitovce, and Štikovo, Tepljuh
	Promina		943	Oklaj, Razvođe, Lukar, Suknovci, Marasovine, Matasi, Puljani, Ljubotić, Čitluk, Mratovo, Bogetić, Podi, Nećven, and Bobodol
	Šibenik		6625	Brnjica, Čvrljevo, Goriš, Gradina, Lepenica, Mravnica, Perković, Podine, Radonić, Vrsno, Danilo, Danilo Biranj, Danilo Kraljice, Boraja, Sitno Donje, Slivno, and Konjevrate
	Total		90,282	

* Seven towns were excluded (Imotski, Omiš, Sinj, Trilj, Vrgorac, Drniš, and Šibenik), and only their rural parts were accounted for.

## Data Availability

The data matrix was deposited in the scientific data repository of Rzeszów University: https://rdb.ur.edu.pl/workflowitems/14/view (accessed on 15 March 2024).
